# A multisystem syndrome compatible with systemic lupus erythematosus: Case report and review of literature

**DOI:** 10.22088/cjim.12.0.482

**Published:** 2021

**Authors:** Samira Alesaeidi, Morteza Daraei, Amir Salami Khanshan, Hamed Zainaldain

**Affiliations:** 1 Department of Internal Medicine, Rheumatology Research Center, Tehran University of Medical Sciences, Tehran, Iran; 2 Amir-Alam Research Center, Tehran University of Medical Sciences, Tehran, Iran; 3 Department of Internal Medicine, Imam khomeini Hospital, Tehran University of Medical Sciences, Tehran, Iran; 4 Student Research Committee, Faculty of Medicine, Iran University of Medical Sciences, Tehran, Iran; 5 School of Medicine, Tehran University of Medical Sciences, Tehran, Iran

**Keywords:** Systemic lupus erythematosus, Lupus nephritis, Cyclophosphamide therapy

## Abstract

**Background:**

Abdominal pain is a routine symptom. Mesenteric arteritis, intestinal vasculitis, enteric vasculitis, mesenteric vasculitis, lupus peritonitis, and abdominal serositis are the possible differential diagnoses. Therefore, lupus enteritis has an uncertain outbreak.

**Case Presentation:**

A 27-year-old woman presented with clinical presentation of peritonitis suggestive of acute abdominal crisis with three days history of fever, bloody diarrhea, nausea, vomiting and seizure. Further work up revealed microangiopathic hemolytic anemia, thrombocytopenia, proteinuria, polyserositis and her initial autoimmune panel all were negative. Since SLE was at the top of our diagnosis, we considered glucocorticoid and cyclophosphamide pulse therapy. After approximately two months of her initial presentation, when all of her symptoms subsided by initial therapy, her antinuclear antibody became positive at 1:320 titers and renal biopsy was compatible with lupus nephritis (stage III).

**Conclusion:**

It is crucial to take the diagnosis of lupus into consideration, in case of any young female with multiorgan involvement even without positive antibody tests. As in this case, it took more than two months after initial presentation to confirm the diagnosis via renal biopsy and only after then, serum autoantibodies became seropositive.

One hundred and 400 people per 100,000 Caucasian and African-Americans are diagnosed with SLE. The ratio of female to male is reported to be 10 to 15 times (1). Abdominal pain is a common symptom in patients with SLE (2). Mesenteric arteritis, intestinal vasculitis, enteric vasculitis, mesenteric vasculitis, lupus peritonitis, and abdominal serositis are the differential diagnosis (3). Therefore, lupus enteritis has an uncertain outbreak (4). 

The involvement of the kidney in systemic lupus erythematosus (SLE), also known as lupus nephritis (LN) is another relatively common serious complication. About 90% of patients with LN are at an unresponsive disorder in renal function. Rapid diagnosis and treatments are vital for patients SLE is a chronic autoimmune disease. SLE can affect each organ. Diagnosis is given after investigation of clinical, laboratory and pathological findings (1). The presenting case is an example of rapidly progressive multisystem SLE which was seronegative and came to hospital with abdominal symptoms, however, she was successfully diagnosed and managed in appropriate time.

## Case presentation

**Figure 1:**
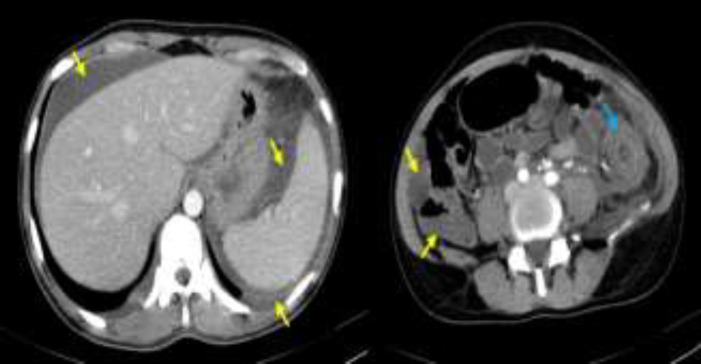
Abdominopelvic computed topography. The yellow arrows indicate ascites and the blue arrow shows the thickening wall of the descending colon compatible with colitis

**Figure 2:**
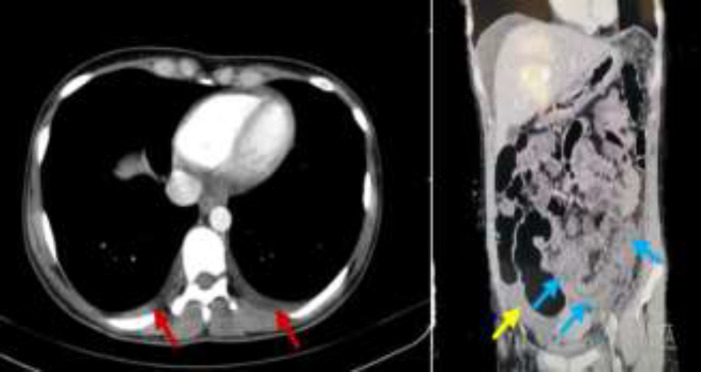
Spiral chest and abdominopelvic computed topography. The red arrows indicate mild bilateral pleural effusion, the yellow arrow shows the ascites in the Douglas pouch and the blue arrows point to the thickening wall of sigmoid and parts of the descending colon

A 27-year-old woman with a history of thalassemia minor was admitted to surgical ward due to clinical presentation of peritonitis suggestive of acute abdominal crisis with three days history of fever, bloody diarrhea, nausea and vomiting. At presentation, she looked pale without icterus and initial vital signs include: blood pressure of 135/85 mmHg, pulse rate 100 beats/minute, Respiratory rate 32 breaths/minute and axillary temperature 39C. Abdominopelvic computed tomography showed no signs of perforation or obstruction but there was prominent thickening of colonic wall from splenic flexure to rectum- compatible with colitis and evidence of ascites and bilateral pleural effusion (figure 1 and 2). Peritoneal fluid paracentesis showed light red aspirate with 550/µl white blood cells (70% polymorphonuclears) and 9000/µl red blood cells. 

**Figure 3:**
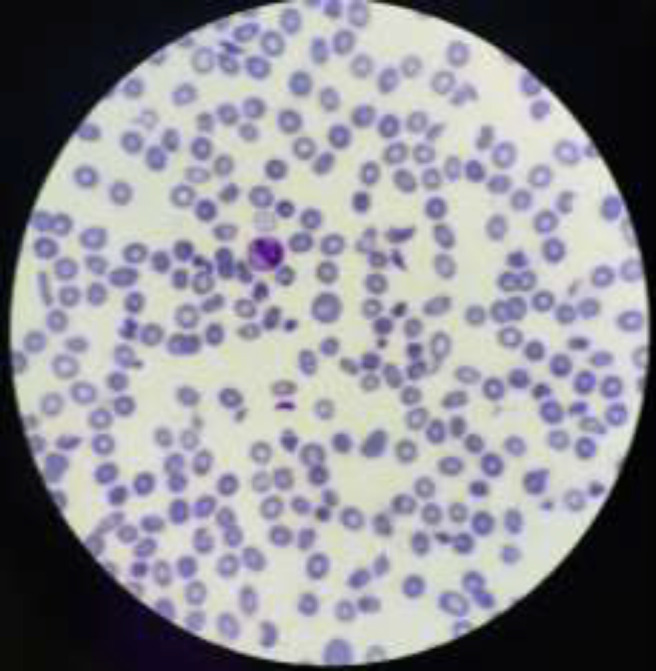
Peripheral Blood Smear shows abundant number of burr cells, thrombocytopenia, target cells and prominent number of schistocytes and fragmented RBCs

Laboratory data showed the evidence of microangiopathic hemolytic anemia (MAHA) with considerable schistocytes (figure 3) and thrombocytopenia. During evaluation, she experienced an episode of tonic-clonic seizure. She referred to internal medicine ward to evaluate thrombotic thrombocytopenic purpura/ hemolytic uremic syndrome (TTP/HUS).

During our physical examination the patient was ill, had periorbital and generalized 2+ pitting edema and was anuric. Further evaluation revealed 4+ proteinuria, rise in plasma creatinine and pericardial effusion (beside ascites and pleural effusion). Her immunologic markers of ANA, anti-dsDNA, Anti-Ro, anti-La and Coombs study were negative and her complement levels showed C_3 _of 77 ng/dl (normal range: 90-190) and normal C_4_ and CH_50_. (the lab data are given in table 1). 

As she had uremic signs, we commenced her hemodialysis with left femoral catheter (only for two successive sections) and with the diagnosis of seronegative SLE, we commenced her intravenous cyclophosphamide and 3 successive days of methylprednisolone pulse. She dramatically responded to treatment but on the 4^th^ day, she developed left common femoral vein thrombosis, therefore anticoagulant started and hemodialysis catheter removed. Antiphospholipid antibody panel was negative. 

We performed a renal biopsy and sent her home. Tissue sample was compatible with stage III lupus nephritis (according to WHO classification) and the ANA became positive with 1:320 titer.

**Table 1:** Clinical laboratory data of the patient

Variable	Normal range	1^St^ day	2^nd^ day	3^rd^ day
**Sodium (meq/l)**	135-145	125	140	136
**Potassium (meq/l)**	2.5-5	3.6	3	35
**Magnesium (mg/dl)**	1.8-2.5	1.6		
**Urea (mg/dl)**	21-52	37	50	63
**Creatinine (mg/dl)**	0.8-1.3	1.6	1.9	4.3
**Fasting Blood Sugar (mg/dl)**	70-99	102		
**Aspartate aminotransferase (U/l)**	50-250	73		
**Alanine aminotransferase (U/l) **	50-250	67		
**Alkaline phosphatase (U/l)**	50-250	78		
**Bilirubin (total/direct) mg/dl**	<1.5-0.3	0.7-0.3	0.8-0.4	
**Lactate dehydrogenase (U/l)**	<480	2082	3657	
**TIBC (µg/dl)**		317		
**Ferritin (ng/l)**	<73	603		
**Total protein (gr/dl)**	6-7.8	3.9		
**Albumin (gr/dl)**	3.5-5.2	2.5		
**Beta-HCG**		Negative		
**Amylase (IU/l)**	<100	47		
**White blood cell count (/µl)**	4-10x10^3^	10600	7100	
**Lymphocyte (%)**	20-40	9		
**Neutrophil (%)**	40-70	88		
**Red blood cell count (/µl)**	4.2-5.4x10^6^	3.9x10^6^	3.37x10^6^	
**Hemoglobin (gr/dl)**	13-17	8.9	7.3	
**Mean corpuscular volume (fl)**	81-99	73	7.3	
**Platelet count (/µl)** **Reticulocyte count**	150-400x10^3^	94 1.3	50	
**Anti HAV-IgM**		Negative		
**Hbs-Ag**		Negative		
**Anti-Hbs Antibody**		>10		
**Anti-HIV**		Negative		
**Anti-HCV**		Negative		
**TSH**	0.5-5	2.5		
**T**_4 (_ **µg/dl)**	4-12.5	3.5		
**Prothrombin time (sec)**	11-15	12.9		
**International normalized ratio**	0.9-1.2	1.2		
**Partial thromboplastin time (sec)**	25-40	28		
**Blood gas ** **PH** **Pco**_2 _ **(mmHg)** **HCO**_3 _ **(mmHg)**	7.36-7.44 35-45 22-26	7.39 30 19	7.28 34 17	

## Discussion

Most, but not all, SLE patients have detectable serum antinuclear antibody (ANA) called ANA-positive SLE, but about 2-3% of cases lack ANA and consider seronegative SLE (5). Many seronegative SLE patients have anti-double stranded DNA, anti-single stranded DNA, anti-Ro or anti-La in their sera but lacking any of them, is also possible (6, 7). CT scan is recognized as a golden standard for the diagnosis of intestinal involvement in SLE disease. Common symptoms of intestinal involvement in SLE patients include an increase in the thickening of the intestinal wall, intestinal dilatation, mesenteric arterial involvement with increased visibility of the vessels (Comb sign) and ascites (8). In this case which was introduced, CT scan was done and there was evidence of intestinal involvement in the CT scan that led us to SLE. Lupus enteritis can be reversed and it responds well to steroid therapy which indicates the need for steroid therapy in the present patient (4).

Given the prevalence of mortality in SLE renal involvement, negative serologic patients with LN are an important challenge for rapid diagnosis and treatment (9). In the case that we reported, this happened. The patient responded well to the steroid therapy (seizure stopped without anti-convulsive therapy, platelet count started to rise, abdominal pain resolved, urine output gradually established and more importantly, her general condition improved dramatically). 

**Figure 4:**
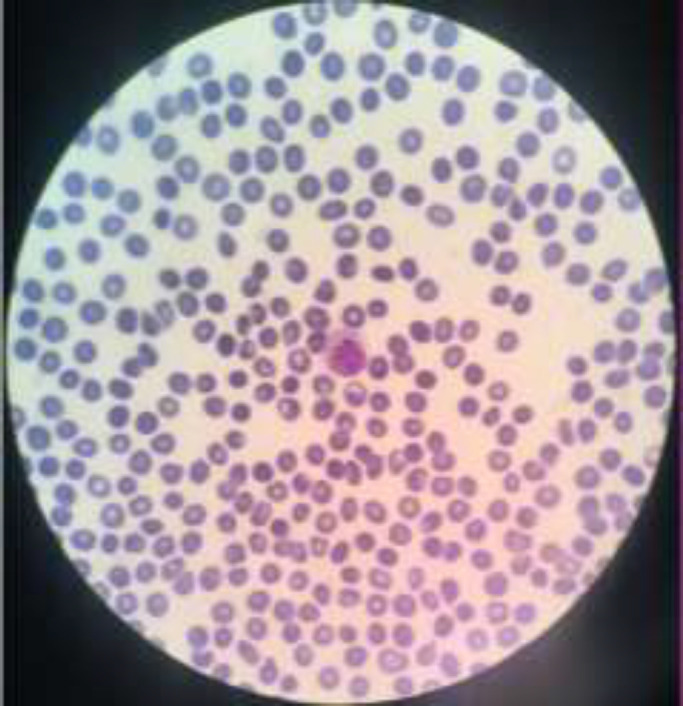
Pripheral Blood Smear after 6 months of treatment

In the literature review, only a few patients were reported to be serologically negative, either with renal or non-renal involvements of the disease (1, 3, 10). One of these patients was followed-up for two months, the other two years and the third had no follow-up. Due to the rare nature of this condition, it is important to follow-up the serology of these patients. Because serology may turn positive in the next follow-ups. Positive serology may take up to 10 years. Also, due to the nature of SLE, autoantibodies are used to detect SLE (6). This was also true for our patient. After 6 months follow-up sessions, she is in her best health condition, her renal function test is normal and her peripheral blood smear is shown in figure 4. 

Although many of her symptoms could occur in TTP but polyserositis and acute kidney injury with heavy proteinuria are considerably unusual for this diagnosis. The most important point in seronegative SLE patients is diagnostic problems that could be easily missed and delay treatment causes devastating outcomes. 

In conclusion any young female with multiorgan involvement, even without positive antibody tests we should take the lupus diagnosis into consideration. As in this case, it took more than two months after the initial presentation to confirm the diagnosis by renal biopsy and only after then, serum autoantibodies became seropositive. Moreover, physicians should avoid CT scanning with contrast in particular patients with polyserositis, proteinuria, hemolytic anemia, and acute renal failure as there is high risk for renal shut down with imaging examination. 
